# Outcome of thrombolysis with streptokinase in patients with prosthetic valve thrombosis

**DOI:** 10.12669/pjms.37.2.3226

**Published:** 2021

**Authors:** Shahid Abbas, Naeem Hameed, Shahbaz Ahmed Khilji, Anjum Jalal

**Affiliations:** 1 Shahid Abbas, FCPS (Cardiology). Associate Professor of Cardiology, Faisalabad Institute of Cardiology, Faisalabad, Pakistan; 2 Naeem Hameed, FCPS (Cardiology). Senior Registrar Cardiology Faisalabad Institute of Cardiology, Faisalabad, Pakistan; 3 Shahbaz Ahmad Khilji, FCPS (Cardiac Surgery). Associate Professor of Cardiac Surgery, Faisalabad Institute of Cardiology, Faisalabad, Pakistan; 4 Anjum Jalal, FCPS-CS, FRCS-CTh, Professor of Cardiac Surgery, Faisalabad Institute of Cardiology, Faisalabad, Pakistan

**Keywords:** Anticoagulation, Prosthetic Valve Thrombosis, Rheumatic Heart Disease, Thrombolysis, Thromboembolism

## Abstract

**Objective:**

To evaluate the outcome of thrombolysis in patients of prosthetic valve thrombosis.

**Methods:**

This retrospective analysis was conducted on data of 84 patients of prosthetic valve thrombosis who presented to emergency room of Faisalabad Institute of Cardiology between July 2017 to December 2019. The diagnosis of prosthetic valve thrombosis was based on clinical suspicion and bed side transthoracic echocardiography done by a consultant cardiologist. Fluoroscopy was done to confirm the diagnosis by observing immobile valve leaflet. The confirmed patients were then treated in emergency with streptokinase after taking an informed consent. Quantitative variables like age were summarized by mean and standard deviation. Qualitative variables like gender, successful thrombolysis, stroke, major bleeding, mortality or re-do surgery were summarized by frequency and percentage.

**Results:**

Mean age was 29 ± 6.36, years and there were more female patients (n=43, 51.25%) as compared to males (n=41, 48.8%). Among the 66 surviving patients thrombolysis was successful without any complications in 56 patients (66.7%). Thrombolysis was successful with minor complications in six patients (7.1%) and it failed to produce desired results in four patients (4.8%). In this study 18 (21.4%) patients died. The common complications included minor bleeding in four patients (4.8%) and major bleeding in 10 patients (12.0 %).

**Conclusion:**

Thrombolysis produces reasonable success rate in cases of prosthetic valve thrombosis who are in functional class I or II. However, it has very high mortality rate in patients presenting with functional class III and IV.

## INTRODUCTION

Prosthetic valves used for better quality of life in patients having valvular heart disease can also have implications on life later on in terms of complications. Major complications include prosthetic valve thrombosis, pannus ingrowth, infection and structural valve deterioration (SVD). Rheumatic valve disease is the commonest reason for valve replacement in developing countries while the other causes including bicuspid aortic valve and ischemic heart disease. Mitral valve is most common valve to be replaced followed by aortic, tricuspid and pulmonary valve respectively.

Patients with mechanical valves need life time anticoagulation whereas the patients with bio-prosthetic valves are kept on anticoagulation only for first three months unless there is any other indication for long term anticoagulation. Prosthetic valve thrombosis is common complication of prosthetic valves. The incidence of mechanical valve thrombosis is estimated at 0.3% to 1.3% per patient-year in developed countries, but as high as 6% per patient-year in developing countries.^1^ In developing country like India, left-sided prosthetic valve thrombosis is noted to be 6.1% of patients within six months of valve replacement surgery.^2^ The main cause of this life-threatening complication is inadequate anticoagulant therapy as the meta-analysis of various studies show that incidence of thromboembolism was increased if warfarin was not taken and in such cases anti-platelet therapy fails to prevent it.^3^ Clinical suspicion of valve thrombosis must be raised when a patient having prosthetic valve presents with embolism, dyspnea, arrhythmias, or cardiogenic shock.^4^ Factors predisposing to thrombus formation are endothelial, hemodynamic and hemostatic. Hemodynamic factors include both hemodynamic characteristics of the prosthesis, as well as overall cardiac hemodynamic status. Localized regions of turbulent flow can develop and lead to stasis and thrombus formation.

Early diagnosis and treatment is necessary owing to high morbidity and mortality of prosthetic valve thrombosis. Surgery was the only treatment till the first successful thrombolysis was reported in a case of tricuspid valve thrombosis in 1971.^5^ Following this report intravenous thrombolysis has become an alternative treatment to surgery in the recent past. The selection of cases for thrombolysis vs surgery is a difficult task. Cine fluoroscopic, transthoracic echocardiography (TTE) and trans-esophageal echocardiography (TEE) are the main modalities used for diagnosis. The predictors of successful thrombolytic therapy include New York Heart Association (NYHA) functional class, level of obstruction, and thrombus size. The TEE is can play a major role in determining the clot burden which is an independent predictor of outcome.^6^ Thrombolytic therapy increases the risk of bleeding and stroke. Despite successful treatment with thrombolytic therapy, up to a third of patients may redevelop prosthetic valve thrombosis in the future.^7^ The European Society of Cardiology recommends thrombolytic therapy only if the risk of surgery is prohibitive or in case, it is not available or the patient cannot be transferred for surgery.^8^ Thrombolytic therapy is becoming the first-line treatment in most of the developing world because several factors including limited availability of surgical expertise, high cost of surgery and patient preference for non-surgical option. It is therefore prudent to evaluate the outcome of thrombolytic therapy which is the main aim of this study.

## METHODS

After approval from hospital Ethical Committee of Faisalabad Institute of Cardiology (# 01-2020/DME/FIC/FSD dated 11-07-2020), this retrospective analysis of database was conducted at Faisalabad Institute of Cardiology (FIC), including patients who underwent thrombolysis for prosthetic valve thrombosis from July 2017 to December 2019. A total of 84 patients were found in our record who underwent thrombolytic therapy in emergency department of FIC. The diagnosis of prosthetic valve thrombosis was suspected clinically in patients with new onset dyspnea of two weeks or less in duration and TTE was done by a consultant cardiologist. Definitive diagnosis was made by observation of immobile valve leaflets on cine fluoroscopy. The TEE was done if transthoracic echo or fluoroscopy were inconclusive.

All patients of age 15 to 70 years, with proved obstructive valve on echo and fluoroscopy were included in study. These included both pregnant or non-pregnant females, single or double valve replacement and also those who had combination of valve replacement and coronary artery bypass grafting (CABG). The exclusion criteria included history of intracranial neoplasm, thrombolysis within last six months, hypersensitivity to streptokinase, asymptomatic non obstructive valve thrombosis and non-thrombotic increase in trans-valvular gradient on TEE.

All eligible patients were provided detailed counseling regarding choices of treatment. Those who consented, were treated with streptokinase and included in the study. Demographic data along with time since valve replacement, type of surgery, type of valve used, compliance to medication, record of INR, and NYHA class at presentation were also noted. Thrombolysis was done either with slow infusion of 250,000 units over 30 minutes followed by 100,000 units/hour for 48 to 72 hours or with accelerated infusion of 500,000 units over 20 minutes then 150,000 units/hour for 10 hours. Successful thrombolysis was defined as complete normalization of valve function i.e. normalization of gradients on echo and normal leaflet motion on fluoroscopy while partial response was defined as >50% reduction in gradients but restricted leaflet motion. Failure of thrombolysis was declared if there was <50% reduction of gradient along with restricted leaflet motion. Major bleeding was defined as gastrointestinal bleeding, intracranial bleeding or any bleeding requiring blood transfusion. The result of thrombolysis was followed by transthoracic echo and cine fluoroscopy. In hospital outcomes were noted on a prescribed Performa.

Data was analyzed by SPSS-25.0 software. Quantitative variables were summarized by mean and standard deviation. Qualitative variables were summarized by frequency and percentage. The outcomes were stratified considering the effect modifiers such as age and gender. Post stratification chi-square test applied. P value ≤ 0.05 considered significant.

## RESULTS

A total of 84 patients presented with 1^st^ episode of prosthetic valve thrombosis and underwent the procedure. Their mean age was 29 years with standard deviation of 6.36 years. There were more female patients (n=43, 51.25%) as compared to males (n=41, 48.8%). Sixteen patients (19.04%) presented in NYHA class III or IV while 68 (80.95%) patients were in class I and II. The baseline characteristics of the patients are presented in Table-I. At the time of presentation, the recent INR was sub optimal in 56 patients. Out of these 56 patients, eight patients (9.52%) had INR of less than 1.5. It was within the range of 1.5 to 2.0 in 28 (33.32%) patients and was between 2 to 2.5 in 20 (23.8%) patients. Only 15 (17.85%) patients had INR in therapeutic range of over 2.5 while 13 (15.47%) patients did not know about their INR in the recent past. The vast majority of the patients were either uneducated (n=25, 29.75%) or received education up to primary level (n=42, 49.98%). Only 16 (19.04%) patients had education up to middle standard while only one patient had completed his education up to matriculation. There was no patient with education level above matriculation which heralds a strong relationship between lack of education and prosthetic valve thrombosis. The regular consumption of green leafy vegetables is frequently incriminated for suboptimal INR but in this study only four patients (4.8%) gave a history of regular use of green leafy vegetables. Interaction with other drugs was also studied and it was found that two patients had taken some antibiotics in last two weeks, four patients had taken NSAIDs off and on and one patient was taking a multi vitamin preparation while 77 (91.6%) patient did not take any other medicine concomitantly which could result in any untoward interaction with warfarin.

**Table-I:** Patient Characteristics.

*Variable*	*Description*	*N*	*%*
Gender	Male	41	48.8
	Female	43	51.2
	Uneducated	25	29.8
Education	Primary	42	50
	Middle	16	19.04
	Secondary or above	1	1.2
Anticoagulation	Optimal (INR >2.5)	15	17.9
	Suboptimal: INR up to 2.5 or Unknown	69	82.1
NYHA class	Class I	1	1.2
	Class II	15	17.9
	Class III	37	44
	Class IV	31	36.8
Operation	MVR	68	81
	DVR	15	17.9
	AVR	1	1.2
Affected Valve	Mitral	80	95.2
	Aortic	4	4.8
	St. Jude	71	84.5
Type of prosthesis	Medtronic	4	4.8
	Carbomedics	4	4.8
	Unknown	5	5.9

Majority of the cases (n=80; 95.2%) were given conventional slow infusion of streptokinase. The accelerated infusion was given in 4 (4.8%) patients. All of them were of class IV dyspnea. Survival was achieved in only one of these four patient.

The outcome in terms of survival and success of thrombolysis and data is stratified according to the NYHA class is shown in Table-II. There was 21.4% mortality (n=18) of prosthetic valve thrombosis undergoing thrombolysis, in our study. Among the 66 (78.6%) patients who survived, 4 (4.8%) patients failed to have desired results of thrombolysis and were referred for surgery. In 56 (%) patients, thrombolysis was successful without any complications. Thrombolysis was successful with minor complications including minor bleed, raised creatinine, cardiac failure in 6(7.2%) patients.

**Table-II:** Comparison of Outcome on the Basis of Functional Class.

*Outcome*	*NYHA I & II (N=16)*	*NYHA III & IV (N=68)*	*All Patients (N=84)*	

	*N*	*N*	*N*	*%*
Survival	14	52	66	78.6
Successful thrombolysis without any complication	8	48	56	
Successful thrombolysis with minor complication	2	4	6	
Failed thrombolysis	4	0	4	
Deaths	2	16	18	21.4

The break-up of bleeding complications is shown in Table-III. It is obvious that bleeding from gastrointestinal tracts is the predominant form of major bleeding and was found in 9 (10.7%) patients. Unfortunately, none of these patients survived.

**Table-III:** Bleeding Complications of Thrombolysis.

*Bleeding Complications*	*NYHA I & II (N=16)*	*NYHA III & IV (N=68)*	*All Patients (N=84)*	

	*N*	*N*	*N*	*%*
Major Bleeding:				
Gastrointestinal	1	8	9	10.7
Intracranial	0	1	1	1.2
Minor Bleeding				
From Teeth, Gums, Oral cavity	2	1	3	3.6
From wound	0	1	1	1.2
Total	3	11	14	16.7

The impact of preoperative counseling regarding anticoagulation was also studied. As given in Table-IV, 62 (74.4%) received proper counseling related to anticoagulation while it was lacking in 22 (26.4%) patients. It appears that the counseling could not prevent lack of compliance as 37 (44.4%) patient missed their warfarin out of 62 patients who received proper counseling while 4 (4.8%) missed warfarin among 22 patients who did not receive counseling. It is obvious from Table-IV that the main reason for missing the tablets in both groups was non-availability of warfarin. That highlights the importance of delivering the warfarin to the door steps of these deserving patients.

**Table-IV:** Compliance vs Counseling regarding Anticoagulation.

*Compliance of Warfarin intake*	*Counseling about Anticoagulation*	

	*Done Properly before & after surgery (n=62)*	*Not done properly (n=22)*
Did not miss tablets	35	18
Missed due to busy schedule	8	0
Missed due to non-availability	9	2
Missed due to poor understanding	1	1
Missed due to side effects	1	0
Missed due to fears	0	0
Missed without any reason	8	1

## DISCUSSION

With improvement in surgical techniques, skills and better post-op care in addition to better understanding of disease and its treatment, prosthetic valves are increasingly used as treatment of valvular heart disease. Developing countries have high prevalence of RHD with most common involvement of Mitral valve.^9^ These patients with symptomatic severe valvular disease undergo valve replacement preferably with metallic valve. The prevalence of PVT is high (6.1%) in developing countries probably due to lack of education and difficulties encountered in access to healthcare.^10^ Majority of patients suffering from PVT are found to have inadequate anticoagulation. We explored the reason behind this suboptimal INR. It was found to be multifactorial including lack of sensitivity about anticoagulation, unavailability of warfarin at certain occasions, inability to monitor INR due to lack of access to nearby healthcare facility, economic reasons and defiance without reason.

The PVT is serious complication of prosthetic valves with high mortality depending on thrombus burden and functional class of patient. Two possible treatment options either thrombolysis or re-do surgery are used as per guidelines. The re-do operations are generally done in patients with high thrombus burden or higher NYHA status i.e. Class III/IV. Thrombolysis is used when expected surgical mortality is high or surgery is unavailable or there is some contraindication to surgery. Thrombolysis may also be used as treatment of choice in patients with small thrombus and good functional class. Guidelines recommend the use of Tissue Plasminogen Activator in preference over streptokinase. The streptokinase can be used both as accelerated infusion or slow infusion.^1^ In one study conducted in 2009, it was found that accelerated infusion has complete response rate of 64.4% whereas slow infusion was 53.3% successful.^11^ However, slow infusion is still used preferentially by many centers due to lesser risk of complications (15%) compared to that in accelerated infusion (18%). At our center 80(95.2%) patients were given slow infusion. Accelerated infusion was given to only four patients, who were in NYHA functional class IV expecting better and quicker response of therapy. Among these four patients only one could survive resulting in 75% mortality in these cases. But as these patients were critical at presentation, we cannot comment on less efficacy of accelerated dose. Our overall mortality was 21.4%. This mortality was higher than that quoted in a study conducted by Karthekyan et al^11^ (7%) but lower than another study by Khattak et al^12^ (25.5%). Considering functional class, our study showed 26.47% mortality in NYHA functional class III and IV which was lower than Khattak et al.^12^ who reported 34.48% mortality in this sub-group. We had 19% patients in FC I/II and 81% in FC III/IV.

Successful thrombolysis without complications was found in 66.7% patients while in 7.1% patients thrombolysis was successful but was associated with minor bleeding complication. Success of thrombolysis was comparable to a study conducted by Nagy et al.^13^ There was no embolic complication in our study. Ten patients suffered from major bleeding; nine had bleeding from gastrointestinal tract and one patient had intracranial hemorrhage. Major bleeding was found in 7.1% patient with majority of them from gastrointestinal origin (5.9%). Major bleeding came out to be lethal as no case of major bleeding in our study could survive. Major bleeding complication was higher in our study than reported by Karthekyan et al.^11^ Failed thrombolysis was found in 4.8% patients which was much lower than the occurrence reported by Caceres-Loriga (8.8%).^4^ It is pertinent to mention that most of the deaths in this study followed a gastrointestinal bleeding. Considering the fact that Faisalabad Institute of Cardiology is a dedicated cardiac institute without the on-site services of other specialties, it is plausible to believe that results could have been better if expert gastroenterology services were available on site. As majority of patients are reluctant to go for re-do surgery, the evaluation of the outcome of Re-do surgery and other thrombolytic agents is required to determine the best treatment modality in our region.

Poor compliance to anticoagulation therapy and suboptimal INR remains a major problem in our community. This has also been observed by Sharif-Khan et al from Rawalpindi Institute of Cardiology who reported that 82.86% of the patients either had suboptimal INR or poor compliance with oral warfarin therapy.^14^ Patil et al has also reported that 76.40% patients in their series had either suboptimal INR or poor compliance to warfarin therapy, which indicates that this phenomenon is common across most of the developing nations.^15^

### Limitations of the study

This study is based on the data of one center only where majority of the patients belong to same ethnic background and relatively poor socioeconomic status. Therefore, the results cannot be generalized for the entire country. Moreover, the study has the inherent flaws of a non-randomized retrospective analysis.

## CONCLUSIONS

Thrombolysis remains a preferred choice of treatment by patients of prosthetic valve thrombosis. It has reasonable success rate in patients with NYHA class I and II. As 23.52% patients in NYHA Class III and IV died despite thrombolysis, more studies are needed to compare the outcome of thrombolysis vs re-do surgery in this high risk sub-group.

### Authors’ Contribution:

**SA** conceived, designed and did statistical analysis & editing of manuscript.

**NH, SAK** did data collection and manuscript writing.

**AJ** did Revision and Editing of manuscript.

**NH** takes the responsibility and is accountable for all aspects of the work in ensuring that questions related to the accuracy or integrity of any part of the work are appropriately investigated and resolved.
